# Estrogen receptors α and β and aromatase as independent predictors for prostate cancer outcome

**DOI:** 10.1038/srep33114

**Published:** 2016-09-09

**Authors:** Thea Grindstad, Kaja Skjefstad, Sigve Andersen, Nora Ness, Yngve Nordby, Samer Al-Saad, Silje Fismen, Tom Donnem, Mehrdad Rakaee Khanehkenari, Lill-Tove Busund, Roy M. Bremnes, Elin Richardsen

**Affiliations:** 1 Dept. of Medical Biology, UiT The Arctic University of Norway, Tromso, Norway; 2 Dept. of Clinical Medicine, UiT The Arctic University of Norway, Tromso, Norway; 3 Dept. of Oncology, University Hospital of North Norway, Tromso, Norway; 4 Dept. of Clinical Pathology, University Hospital of North Norway, Tromso, Norway

## Abstract

Androgens are considered important in normal prostate physiology and prostate cancer (PCa) pathogenesis. However, androgen-targeted treatment preventing PCa recurrence is still lacking. This indicates additional mediators contributing to cancer development. We sought to determine the prognostic significance of estrogen receptors, ERα and -β, and the aromatase enzyme in PCa. Tissue microarrays were created from 535 PCa patients treated with radical prostatectomy. Expression of ERα, ERβ and aromatase were evaluated using immunohistochemistry. Representative tumor epithelial (TE) and tumor stromal (TS) areas were investigated separately. Survival analyses were used to evaluate the markers correlation to PCa outcome. In univariate analyses, ERα in TS was associated with delayed time to clinical failure (CF) (p = 0.042) and PCa death (p = 0.019), while ERβ was associated with reduced time to biochemical failure (BF) (p = 0.002). Aromatase in TS and TE was associated with increased time to BF and CF respectively (p = 0.016, p = 0.046). Multivariate analyses supported these observations, indicating an independent prognostic impact of all markers. When stratifying the analysis according to different surgical centers the results were unchanged. In conclusion, significant prognostic roles of ERα, ERβ and aromatase were discovered in the in PCa specimens of our large multicenter cohort.

Prostate cancer (PCa) is continually a challenge as one of the leading causes of cancer-related death amongst men^1^. Androgens are considered as key regulators of physiological processes in the prostate, including prostatic growth, differentiation, development and secretory function, but their role in PCa pathogenesis is not yet defined^2^,^3^. The response to androgens is mediated thorough the androgen receptor (AR), which is expressed in both prostatic epithelial and stromal cells^4^. This androgen-dependency has been thoroughly investigated and formed the basis for androgen deprivation therapy (ADT), which is an essential PCa treatment in metastatic disease. Innovative approaches in androgen signalling targeting are developing. Oral inhibitors targeting CYP-17 (by abiraterone) and the AR (by enzalutamide) has increased survival in metastatic castration-resistant PCa (CRPC) in phase III studies^5^,^6^,^7^,^8^. However, recurrence of CRPC still remains a challenge. This indicates a complexity in the progression from invasive cancer to castration refractory disease and additional mediators appear to be involved in this malignant transformation.

The involvement of androgens in PCa has led to an increased interest in the involvement of other sex steroid hormones and their synthesis in PCa development. Local estrogen production happens thorough the conversion of androstenedione to estrone, and testosterone to estradiol which is catalyzed by the aromatase enzyme (CYP 19). This process takes place in several tissues, including the prostate^9^,^10^,^11^. Aromatase inhibitors are currently used in treatment of advanced breast cancer in post-menopausal women. The effect of aromatase inhibitors on CRPC has also been investigated, however a beneficial effect has not been shown^12^,^13^. So far, results regarding local aromatases activity in PCa have been diverging^9^,^10^,^11^, and few studies have focused on the association between local aromatase expression and PCa. Currently, genetic polymorphism of the aromatase gene, *CYP19A1,* and it’s association to PCa has received interest and is undergoing investigation^14^,^15^,^16^.

The involvement of estrogens in PCa is not a novel concept^2^,^3^. Estrogens were used as the main PCa treatment until the 1950s due to their ability to suppress serum testosterone levels via negative feedback on luteinizing hormone (LH) production^17^. However, as serious cardiovascular side effects were an increasing concern, new ADT methods developed (e.g. LH - releasing hormone antagonists) and estrogen treatment was discarded^17^.

The effects of estrogens are mediated through two different receptors, ERα and ERβ^18^, both expressed in the human prostate. Estrogens involvement in PCa development received renewed interest after the discovery of the second ER receptor (ERβ) in the prostate^18^. This has led to development of a paradigm regarding the different roles of the ERs in PCa. So far the hypothesis has been that ERβ has a predominantly protective effect in PCa, while ERα is oncogenic^19^,^20^,^21^,^22^,^23^. However, the role of ERs in PCa remains controversial as opposing results regarding their behavior in PCa development are still emerging^24^,^25^,^26^,^27^,^28^,^29^,^30^,^31^.

In order to understand the ERs involvement in PCa we have investigated the epithelial and stromal expression of ERα, ERβ and aromatase in different tissue compartments in a large cohort of 535 prostatectomy specimens. We further analyzed their prognostic impact on patient outcome and correlation to clinicopathological variables. All three markers were detected in either tumor related stromal cells (TS), tumor epithelial cells (TE) or both and correlated to PCa outcome.

## Materials and Methods

### Patients and tissue data

Primary tumor tissue from 535 radical prostatectomy (RP) patients was included in this study. The tumor tissue was retrospectively collected from the Departments of Pathology at the University Hospital of Northern Norway (n = 248), Nordland Hospital (n = 59) and St. Olavs Hospital (n = 228) from the period 1995–2005. Patients who had (I) radiotherapy to the pelvic region prior to surgery, (II) other malignancies within 5 years prior to the PCa diagnosis, (III) inadequate paraffin-embedded tissue blocks, and (IV) lack of clinical follow-up data, (V) received hormonal therapy prior to or at the time of the prostatectomy, were excluded. All primary cancers were histologically reviewed by two pathologists (ER and LTB) and the tumors were graded according to the modified Gleason grading system^32^,^33^ and staged according to the WHO guidelines^34^. Median follow-up time of survivors was 89 (range 6–188) months at the last patient update in November 2012. The cohort is thoroughly described in a previous paper^35^.

The Regional Committee for Medical and Health Research Ethics (2009/1393), the Data Protection Official for Research (NSD), and the National Data Inspection Board approved this study. All patients were made anonymous with each trial number. These numbers were initially linked to identity for only one purpose prior; to collect clinical information. Written consent from the patients was considered, but as this was a retrospective study where most of the material was more than 10 years old and most of the patients deceased, it was considered not needed. The aforementioned parties accepted this solution. All data was analyzed anonymously.

### Microarray construction

Tissue Microarray (TMA) construction was chosen for high-throughput molecular pathology analysis. For each case, a pathologist (ER) identified and marked two representative areas of tumor tissue (epithelial tumor cells), two with tumor stromal tissue, one area with normal epithelial tissue, and one area with normal stromal tissue. From each of these areas, cores were sampled from each donor block in order to construct TMA blocks.

The TMAs were assembled using a tissue-arraying instrument (Beecher Instruments, Silver Springs, MD, USA). We used a 0.6 mm diameter needle to harvest cores from the marked tissue areas from the corresponding formalin-fixed paraffin-embedded (FFPE) tissue blocks. The samples were inserted into an empty recipient paraffin block according to a coordinate pattern. To include all core samples, twelve tissue array blocks were constructed. Multiple 4 μm sections were cut with a Micron microtome (HM355S), affixed to glass slides, and sealed with paraffin. The detailed methodology has been reported previously^36^.

### Immunohistochemistry (IHC)

**Figure 1:**
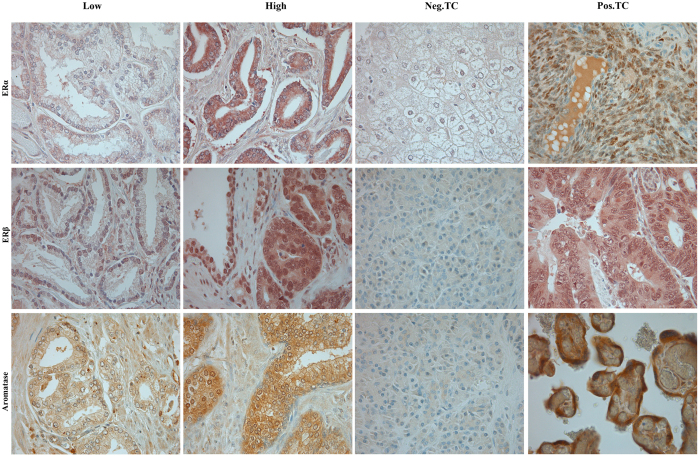
Immunohistochemical analysis of estrogen receptor (ER)α, ERβ and aromatase in prostate cancer (PCa) specimens and tissue controls. Microscopic pictures of tissue micro array representing expression of aromatase, estrogen receptor (ER)α and ERβ by immunohistochemistry staining in PCa sections. Original magnification x40 showing low and high expression of ERα, ERβ and aromatase in in tumor cells (TE) and tumor associated stromal cells (TS) of PCa in addition to positive tissue controls (Pos.TC) and negative tissue controls (Neg.TC) for each antibody. Positive tissue controls; ERα – ovary, ERβ –colon adenocarcinoma and aromatase – placenta. Negative tissue controls; ERα – liver, ERβ and aromatase – pancreas.

The following antibodies were used in this study: Rabbit polyclonal ERα antibody (SC-543, Santa cruz, 1/100), mouse monoclonal ERβ antibody (clone PPG5/10, MCA1974s, AbD Serotec, 1/10), and goat polyclonal aromatase (CYP-19) antibody (SC-14245, Santa cruz, 1/50). The TMA slide sections were deparaffinised and rehydrated and antigen retrieval was performed by microwaving (450 W) in 0.01 M citrate buffer at pH 6.0 for 20 minutes. The sections were cooled to room temperature (RT) and endogenous peroxidase activity was blocked by incubation with a solution of 0.5% hydrogen peroxide for 10 minutes. The sections were then incubated in 5% normal serum ABC kit (Vector Laboratories) for 1 h at RT to block nonspecific binding. Subsequently, the sections were incubated overnight at 4 °C with primary antibodies, however for goat polyclonal aromatase the incubation time was 45 minutes at RT. After washing, the sections were incubated with the corresponding secondary antibodies for 1 h at RT. The Vectastain ABC kit (Vector Laboratories) was used for the avidin-biotin complex method according the manufacturer’s instructions. The sections were lightly counterstained with hematoxylin, dehydrated through an ethanol series, cleared in xylene and mounted. Two different controls for our staining method were applied. Firstly, control staining of the sections with an isotype-matched control antibody without the primary antibody. Secondly, multiple organ tissue microarray as positive and negative tissue controls were used to verify the specificity of the staining in every staining procedure. The positive tissue controls comprised ovary for ERα, colon adenocarcinoma for ERβ and placenta for aromatase; Negative tissue controls were samples of normal pancreas and liver. Details regarding antibody validation are presented in supplementary information (S1) and IHC staining of control tissue is depicted in Fig. 1.

### Scoring of IHC

The ARIOL imaging system (Applied Imaging Corp., San Jose, CA, USA) was used to scan and digitalize the IHC stained TMA slides. The slides were loaded in the SL 50 automated slide loader and scanned at a low resolution (1.25x) and high resolution (20x) using an Olympus BX61 microscope with an automated platform (Prior Scientific, Cambridge, UK). Images of the cores were uploaded into the ARIOL Software. All samples were de-identified and scored manually by two experienced parties independent of each other: ERα and ERβ by two pathologists (ER and SFI) and aromatase by one pathologist (ER) and one MD student (TG) trained by an experienced pathologist. Consequently, all reported marker expressions are based on two separate evaluations of the tissue cores. The scoring was done semi-quantitatively and both parties were blinded to any pathological or clinical information. In case of discrepancy of more than one, the slides were re-examined. When selecting the representative pictures of IHC stained TMA cores depicted in Fig. 1, the TMA slides where evaluated by microscope and pictures of the selected cores were taken manually through microscope.

Overall, the percentage of ERα and ERβ positive cells varied between the different cores, there was however little variation in staining intensity. ERα and ERβ density was therefor given a score between 0–3, reflecting the percentage of positive cells in the examined compartment. The applied scoring system was as follows: 0: 0%, 1: ≤5%, 2: 6–50%, 3: >50%. For aromatase there was an overall high percentage of positive cells, but variation in staining intensity was observed. The degree of aromatase protein expression in cytoplasm was therefore graded according to the dominant staining intensity. The scoring was done using the following system: 0 = negative, 1 = weak, 2 = moderate, 3 = strong. For each case, mean scores were calculated. Further, the scoring values were dichotomized as high and low intensity or density of stained cells. Both median, mean and quartile cut off values were considered, but the optimal cut off was chosen based on adequate number of patients in each group and statistical trends. The cut off for ERβ in TS and aromatase in TE was defined as the median (1.5, 1.0) value. For ERα and aromatase in TS the cut off was set to the value ≥1^st^ quartile (0.75, 0.63). Marker expressions were evaluated in all the different PCa compartments: Normal epithelia (NE), normal stroma (NS), hyperplasia (H), TE and TS. Further, marker expression in the different compartments and their correlation with biochemical failure (BF), clinical failure (CF) and prostate cancer death (PCD) was analyzed.

### Statistical methods

All statistical analyses were performed using the statistical package IBM SPSS, version 21 (SPSS Inc., Chicago, IL, USA). A Wilcoxon signed rank test was used to assess differences in ERβ, ERα and aromatase expression between the different compartments: TE vs. TS. Spearman correlation coefficient was performed to examine the association between ERβ, ERα, aromatase expression and clinicopathological variables. The Kaplan-Meier method was used for the univariate survival analysis, and log-rank test was used to assess statistical significance. Univariate analyses were constructed for the following end-points: (1) Biochemical failure free survival (BFFS), (2) Clinical failure free survival (CFFS) and (3) PCa death free survival (PCDFS). BF was determined as PSA recurrence ≥0.4 ng/ml in a minimum of two different blood samples postoperatively^37^. CF was defined as verified local symptomatic recurrence and/or findings of metastasis to bone, visceral organs or lymph nodes by CT, MR, bone scan or ultrasonography. PCD was defined as death caused by progressive and disseminated castration-resistant PCa uncontrollable by therapy. All significant variables from the univariate analysis were entered in the multivariate analysis using backward stepwise Cox regression model with a probability for stepwise entry removal at 0.05 and 0.1, respectively. The IHC scoring values from each pathologist were compared for inter-observer reliability by use of a two-way random effect model with absolute agreement definition. The significance level used was p < 0.05 for all analyses.

## Results

### Patient characteristics

An overview of the patient’s demographic, clinical and histopathological characteristics is presented in Table 1. Median age at surgery was 62 years (47 to 76). The radical prostatectomy was retropubic in 435 cases (81%) and perineal in 100 cases (19%). Combined Gleason score ranged from 6-to10 and tumor stage from T2a to T3b. Median PSA was 8.8 (range 0.7–104). At the last follow-up in 2012, 170 (32%) had experienced BF, 36 (7%) experienced CF and 15 (3%) had died due to PCa.

### Scoring agreement

There was a good scoring agreement between the scorers. The intra-class correlation coefficient (reliability coefficient, r) was 0.93 (p < 0.001) for the ERα marker and 0.79 (p < 0.001) for the ERβ marker and 0.89 (p < 0.001) for the aromatase marker respectively.

### Expression of ERα, ERβ and aromatase expression and their correlation with clinicopathological variables

ERα and aromatase staining was predominantly cytoplasmic (Fig. 1). The staining of ERβ was both nuclear and cytoplasmic (Fig. 1). ERα staining in epithelial cells was primarily negative (NE and TE negative in 70 and 64%, respectively) (Fig. 1). For the small selection of patients with a positive epithelial ERα expression, no significant difference in BFFS, CFFS or PCDFS was found. ERβ staining was overall positive in stromal and epithelial cells of both benign and malignant prostate tissue. The percentage of ERβ positive cells was however significantly higher in TE compared to TS (mean value 1.93 and. 1.26 respectively, p < 0.001). Aromatase staining was also in general positive. Though, a stronger aromatase expression was detected in NS compared to TS (mean value 1.29 and 1.09 respectively, p < 0.001). There was also a stronger aromatase staining intensity in NS compared to NE (mean value 1.29 and 1.05 respectively, p < 0.001). No further difference in expression was detected for either marker.

The correlation between marker expressions and clinicopathological variables was weak or non-significant (r < 0.2). However, a positive correlation was detected between ERα and ERβ in TS (r = 0.50, p < 0.001). As expected, in TS both ERα and ERβ displayed a correlation to aromatase (r = 0.36, p < 0.001 and r = 0.53, p < 0.001). The same correlation was observed in TE for ERα, ERβ and aromatase respectively (r = 0.22, p < 0.001/r = 0.43, p < 0.001).

### Univariate analysis

Variables significant for BF were pT-stage (p < 0.001), pN-stage (<0.001), preoperative PSA (p < 0.001), Gleason score (p < 0.001), tumor size (p < 0.001), perineural infiltration (PNI, p < 0.001), positive surgical margin (PSM, p = 0.041), apical PSM (p = 0.040), non-apical PSM (p < 0.001), and lymphovascular infiltration (LVI, p < 0.001). For CF, significant prognostic factors were: pT-stage (p < 0.001), pN-stage (p < 0.001), Gleason score (p < 0.001), tumor size (p = 0.019), PNI (p = 0.001), PSM (p = 0.038), non-apical PSM (p < 0.001) and LVI (p < 0.001). For PCD the prognostic factors were: pT-stage (p = 0.027), pN-stage (p < 0.001), Gleason score (p < 0.001), PNI (p = 0.002), non-apical PSM (p = 0.029) and LVI (p = 0.009).

**Figure 2:**
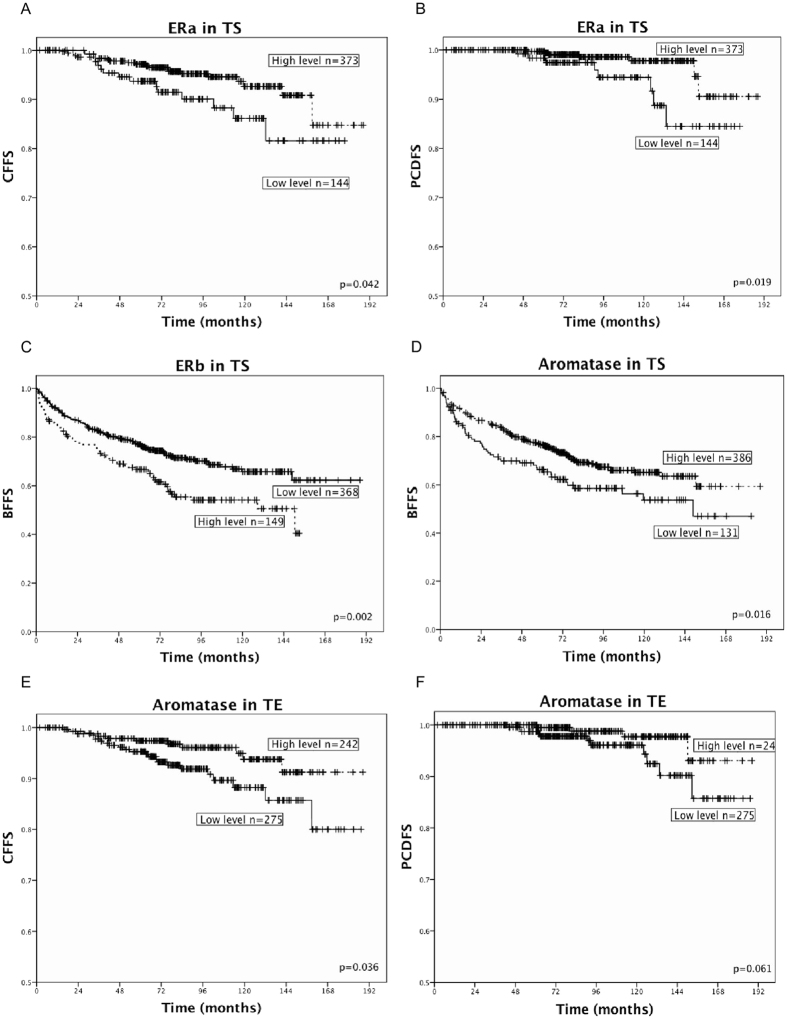
Association with prostate cancer outcome and estrogen receptor (ER) α, ERβ and aromatase expression level. Kaplan Meier curves displaying biochemical failure free survival (BFFS), clinical failure free survival (CFFS) and prostate cancer death free survival (PCDFS) in relation to high or low expression level of ERα, ERβ and aromatase expression in prostate cancer (PCa) patients (n = 535). (**A**) ERα in tumor associated stromal cells (TS) and CFFS. (**B**) ERα in TS and PCDFS. (**C**) ERβ in TS and BFFS. (**D**) Aromatase in TS and BFFS. (**E**) Aromatase in tumor cells (TE) and CFFS. (**F**) Aromatase in TE and PCDFS.

Results from univariate analysis of molecular markers according to BFFS, CFFS and PCDFS are presented in Table 2 and Fig. 2A–F. In TS, a high density of ERα was associated with increased CFFS (p = 0.042) (Fig. 2A) and increased PCDFS (p = 0.019) (Fig. 2B), albeit this trend was not displayed in BFFS (p = 0.819). High ERβ expression was on the other hand associated with reduced BFFS (p = 0.002) (Fig. 2C). Further, a strong TS staining intensity of aromatase was associated with increased BFFS (p = 0.016) (Fig. 2D). In TE, a strong intensity of aromatase was also associated with increased CFFS (p = 0.036) (Fig. 2E) and similar curves tending towards significance were observed for PCDFS (p = 0.061) (Fig. 2F). When stratifying these analyses according to the different surgical centers the same trends were displayed. In addition to these findings, we demonstrate a trend for the markers ERα in TS and aromatase in TE in adding prognostic value (4–12% reduced 10-year CFFS in low versus high expression subgroups) within each pathological PCa stage (Table 3).

When merging the expression levels of ERα and aromatase in TS, a combined high level of the two markers (high/high vs. high/low, low/high, low/low) was associated with increased CFFS (p = 0.029) (S1 Table 2). The same tendency was also displayed when merging ERα in TS and aromatase in TE. A combined high level (high/high, high/low, low/high vs. low/low) was associated with increased CFFS (p = 0.038) and PCDFS (0.003), but not BFFS (p = 0.854) (S1 Table 2). Further, when merging the stromal expression of ERβ and aromatase, a beneficial effect of a combined level low ERβ and high aromatase (low/high) in BFFS stood out compared to the high ERβ and low aromatase (high/low) combination which was associated with reduced time to BFFS (p < 0.001) (S1 Table 2). When combining ERβ in TS and aromatase in TE, no obvious trends or significant results were displayed.

### Multivariate analysis

Results from multivariate analysis are presented in Table 4. In addition to pT-stage, Gleason score ≥ 9 apical PSM, and non-apical PSM, both ERβ (HR: 1.70, 95% CI: 1.19–2.42, p = 0.004) and aromatase (HR: 0.55, 95% CI: 0.38–0.80, p = 0.002) in TS were independent prognostic factors for BF. ERα in TS emerged as a significant, independent marker for CF (HR: 0.43, 95% CI: 0.22–0.86, p = 0.018) in addition to non-apical PSM, PNI and Gleason grade ≥ 9. This was also the case for aromatase in TE (HR: 0.43, 95% CI: 0.21–0.90, p = 0.024). Further, ERα in TS was the only marker that served as an independent prognostic factor for PCD (HR: 0.28, 95% CI: 0.1–0.78, p = 0.015) along with Gleason grade ≥ 9 and PNI, although aromatase in TE tended towards significance. Further, ERα in TS and aromatase in TE combined emerged as an independent prognostic factor for CF (HR: 0.43, 95% CI: 0.21– 0.87, p = 0.02) and PCD (HR: 0.24, 95% CI: 0.085–0.65, p = 0.005). The combination ERα and aromatase and ERβ and aromatase respectively in TS did not reach statistical significance in multivariate analyzes.

## Discussion

In our large cohort of 535 PCa specimens, an independent association was detected between PCa outcome and ERα, ERβ and aromatase expression. In TS, high-density of ERα was independently and significantly associated with both increased CFFS and PCDFS. In contrast, a high ERβ density level was independently and significantly associated with reduced BFFS. Further, a strong staining intensity of aromatase in both TS and TE was significantly and independently associated with increased BFFS and CFFS respectively. In addition, a correlation and an additive effect were discovered when analyzing the combined expression of ERs and aromatase. A major strength of our study is the large multicenter cohort and the long follow-up. In addition, our results were validated in two different cohorts, yielding data tending towards the end results in the total cohort. In addition, few studies have investigated these markers independently in both epithelial and stromal areas of PCa with a clinical event-free survival.

In accord with previous publications, ERα density level was predominantly negative in NE in the PCa patients^23^. However, we did not observe an increased expression of epithelial ERα in TE compared to NE, nor a previously reported correlation between ERα versus Gleason grade or tumor progression^21^,^28^. But notably, patients with high ERα level in TS had significantly increased PCDFS. This is supported by other studies^24^,^29^,^30^,^31^ and indicates a more complex role of ERα in PCa than the previously ascribed role as a tumor promoter. In fact, Slavin *et al.* discovered using IHC, *in vitro* invasion assays and *in vivo* studies that ERα in TS is beneficial for PCa patients^29^. This could potentially be attributed to a PCa metastasis-suppressing role of ERα^29^. In a recent follow-up article, Slavin *et al.*^30^ further hypothesize that ERα in TS of prostate cancer can be utilized as a prognostic marker to predict cancer progression. In addition, Zellweger *et al.* detected an improved overall survival for CRPC patients with stromal ERα expression^24^. This is further supported by Celhay *et al.* who noted survival to be significantly reduced in PCa patients with low stromal ERα expression^31^.

In our material ERβ was expressed, to various extents, in the majority of both epithelial and stromal cells. This is in agreement with previous publications^23^,^24^,^38^, although some report ERβ to be predominantly localized in the basal cell epithelial compartment and to a lesser extent in the stromal. Many reports, including this, suggest a negative role of ERβ expression on PCa prognosis^24^,^25^,^26^,^27^,^28^. There have also been reports of a tumor promoting role of ERβ, especially in PCa metastasis^23^,^25^,^26^. This may indicate ERβ to exert various effects at different stages of PCa development. However, several publications have delivered contradictory reports on the protective role of ERβ in PCa, e.g., loss of ERβ as cancer progresses^20^,^22^,^23^. Supporting our findings, Zellweger *et al.* reported that increased ERβ expression in hormone naïve PCa (HNPC) was associated with a worse outcome^24^. Possible reason for the adverse effect of ERβ has previously been described. Yang *et al.* reported that non-androgenic proliferation of PCa can occur through estrogen-mediated activation of AR in complex with ERβ and proline-, glutamic acid- and leucine rich protein 1 (PELP1), an AR cofactor known for its proto-oncogenic abilities^27^. It has also been reported a correlation between ERβ and Cyclin D1 in hormone-naïve PCa patients^39^, a protein with known proliferative function.

Recently, several ERβ isoforms have been isolated and different functions of these isoforms have been hypothesized, including several with tumor promoting abilities^25^,^26^,^40^. Recent evidence suggests that subtype ERβ2 promotes migration and invasion of cancer cells in addition to cell proliferation, whereas ERβ1 has tumor-suppressing effects^26^. Further evidence suggest ERβ2 to be a functional modulator of ERα and ERβ1^26^. Considering the strong correlation between ER subtypes in TS, the hypothesis of an interaction and regulation between these receptors is strengthened. Our study and several previous publications, has not investigated ERβ isoforms. This is, however, an important topic for future research and could explain some of the previous diverging results regarding ERβ.

Our study demonstrated a wide distribution of aromatase in stromal and epithelial cells of both benign and malignant prostate tissue. Aromatase has previously been detected in both epithelial and stromal tissue, but agreement regarding its compartmental expression is however currently lacking^9^,^10^,^11^. There are limited recent studies investigating the expression of aromatase in various prostatic tissue compartments, with respect to PCa pathogenesis. However, two studies observed a positive association between aromatase and PCa recurrence^31^,^41^, contradicting our findings. Genetic polymorphism in the gene encoding aromatase, *CYP191A,* has also been a topic of interest. There have been reports, however with equivocal results, indicating that different single nucleotide polymorphism (SNPs) in *CYP191A* influences PCa risk and survival^14^,^16^. This association is however disputed by others^15^. It is not evident how increased aromatization can exert a beneficial mechanism in PCa. Besides the role of SNPs and aromatase in PCa, an explanation may be a local depletion of testosterone due to the shuttling of testosterone towards estrogen production. This could in turn decrease stimulation of the AR.

By detecting aromatase expression in the PCa specimens, in addition to the strong correlation between aromatase and the ERs in TS and TE, we confirm a local production of estrogens in PCa and its stimulation of the local receptors. This indicates estrogens’ ability to directly act upon the prostate gland, not only thorough negative feedback on the hypothalamic-pituitary-gonadal axis. This is of particular interest since it is still unresolved whether locally produced or circulating hormones effect PCa more^42^.

There are several factors that may explain some of the discrepancies regarding these hormonal biomarkers. The reproducibility of prognostic biomarker studies is always a challenge^43^. The cohorts are different, the tissue handling and fixation are different, the lab procedures for biomarker detection (in this case IHC) are different and details on intraprostatic localization of scoring and the biomarker expression analyses are different. Considering the extent of discrepancy in the large number of publications available, a systematic review/meta-analysis with subsequent validation of the most promising studies is highly warranted. The heterogenous nature of the prostate, the different downstream responses to stimulation of stromal or epithelial receptors, respectively, the stromal-epithelial interactions, and the crosstalk between the ARs, ERαs and ERβs are all factors complicating attempts to decipher roles of the different sex steroid hormones in PCa pathogenesis. This complexity is demonstrated by contradicting results between human PCa samples^24^,^25^ and PCa cell line studies^11^,^20^,^41^. As an example, several preclinical studies have described protective effects of selective estrogen-receptor modulators (SERM) on PCa through the activation of ERβ^44^,^45^. However, this mechanism has to our knowledge never been effectively adapted in the clinic. This is also the case for studies investigating ERα blockage and aromatase inhibitors^12^,^13^,^46^.

In the present study, ERs and aromatase emerged as potential prognostic biomarkers for PCa in addition to other well-established markers. This is demonstrated by the significant impacts in the multivariate analyses (Table 4). In addition, we observed that our markers added prognostic value (4–12% reduced 10-year CFFS in low versus high expression subgroups) even within each pathological stage (Table 3). With additional confirmation, it is likely that this can be adapted to at least a sub-group of PCa patients in the future.

## Conclusion

We found both ERs and aromatase to be significantly and independently associated to PCa outcome. In TS, a high expression of ERα was associated with increased CFFS and PCDFS, while a high expression of ERβ was associated with reduced BFFS. In addition, high aromatase expression in both TS and TE was favorable with respect to BFFS and CFFS, respectively. For CFFS, the impact of these markers added prognostic relevance within each stage group. This knowledge may be valuable for the development of future prognostic biomarkers in PCa, but further validation is warranted before clinical application.

## Additional Information

**How to cite this article**: Grindstad, T. *et al.* Estrogen receptors α and β and aromatase as independent predictors for prostate cancer outcome. *Sci. Rep.* **6**, 33114; doi: 10.1038/srep33114 (2016).

## Supplementary Material

Supplementary Information

## Figures and Tables

**Table 1:** Patient characteristics and clinicopathological variables as predictors of biochemical failure-free survival, clinical failure-free survival and disease-specific survival (univariate analysis; log-rank test) (N = 535).

Characteristic	Patients (n)	Patients (%)	BF (170 events)		CF (36 events)		PCD (15 events)	
			5-year EFS (%)	p	10-year EFS (%)	p	10-year EFS (%)	p
Age				0.55		0.085		0.600
≤65 years	357	67	76		92		97	
>65 years	178	33	70		88		96	
pT-Stage				**<0.001**		**<0.001**		**0.027**
pT2	374	70	83		96		98	
pT3a	114	21	60		86		98	
pT3b	47	9	43		73		89	
pN-stage				**<0.001**		**<0.001**		**<0.001**
NX	264	49	79		95		98	
N0	268	50	71		89		97	
N1	3	1	0		33		67	
Preop PSA				**<0.001**		0.085		0.061
PSA < 10	308	58	80		93		99	
PSA > 10	221	41	67		88		95	
Missing	6	1	—		—		—	
Gleason				**<0.001**		**<0.001**		**0.001**
3 + 3	183	34	83		98		99	
3 + 4	220	41	76		94		98	
4 + 3	80	15	69		84		95	
4 + 4	19	4	63		76		94	
≥9	33	6	34		67		87	
Tumor size				**<0.001**		**0.019**		0.098
0–20 mm	250	47	82		94		99	
>20 mm	285	53	67		88		96	
PNI				**<0.001**		**<0.001**		**0.002**
No	401	75	79		95		98	
Yes	134	25	60		81		93	
PSM				**0.041**		**0.038**		0.697
No	249	47	81		94		97	
Yes	286	53	69		89		97	
Non-apical PSM				**<0.001**		**<0.001**		**0.029**
No	381	71	81		95		98	
Yes	154	29	57		81		94	
Apical PSM				0.040		0.484		0.313
No	325	61	73		90		96	
Yes	210	39	77		92		98	
LVI				**<0.001**		**<0.001**		**0.009**
No	492	92	77		93		98	
Yes	43	8	46		71		87	
Surgical proc.				0.230		0.414		0.581
Retropubic	435	81	76		90		97	
Perineal	100	19	67		95		98	

Abbreviations: BF = biochemical failure; CF = clinical failure; PCD = prostate cancer death; PCa = prostate cancer; EFS = event free survival; LVI = lymphovascular infiltration; NR = not reached; PNI = Perineural infiltration; Preop = preoperative; PSA = Prostate specific antigen; PSM = Positive surgical margin; Surgical proc = surgical procedure.

**Table 2:** Marker expressions as predictor for BFFS, CFFS and PCDFS in PCa patients (n = 535), (univariate analysis; log rank test), significant p-values in bold (threshold p ≤ 0.05).

		Patients (n)	Patients (%)	BFFS			CFFS			PCDFS		
Marker expression				5-year (%)	10-year (%)	p	5-year (%)	10-year (%)	p	5-year (%)	10-year (%)	p
ERα TS	Low	144	26.9	73	67	0.819	94	86	**0.042**	98	89	**0.019**
	High	373	69.7	74	61		97	93		99	98	
	Missing	18	3.4									
ERβ TS	Low	368	68.8	77	66	**0.002**	97	91	0.658	100	97	0.486
	High	149	27.9	67	54		95	91		99	97	
	Missing	18	3.4									
Aromatase TS	Low	131	24.5	66	54	**0.016**	94	90	0.225	98	91	0.668
	High	386	72.1	77	65		97	91		99	98	
	Missing	18	3.4									
Aromatase TE	Low	275	51.4	73	61	0.487	95	93	**0.036**	98	96	0.061
	High	242	45.2	75	64		97	96		99	97	
	Missing	18	3.4									

Abbreviations: ERα = estrogen receptor alpha; ERβ = estrogen receptor beta; TE = tumor epithelial cells; TS = tumor stromal cells; BFFS = Biochemical failure free survival; CFFS = clinical failure free survival; PCDFS = prostate cancer death free survival.

**Table 3:** Ten year CFFS for patients with low or high levels of ERα in TS and aromatase in TE respectively in relation to prognostic groups of PCa.

Risk groups of localized prostate cancer	10 year CFFS (%)					
	ERα in TS			Aromatase in TE		
	Low (%)	High (%)	p	Low (%)	High (%)	p
I (*n* = *42*)	NE	NE	—	NE	NE	—
IIA (*n* = *109*)	92	96	0.886	92	96	0.904
IIB (*n* = *206*)	87	99	**0.001**	93	97	0.148
III (*n* = *154*)	76	84	0.442	76	88	0.074

The stratification of our cohort into prognostic groups are constructed according to the American Joint Committee on Cancer (AJCC) TNM system. By adding either the ERα or the aromatase marker to the already well-established clinical markers, prognostic impact is added across each pathological stage (univariate analysis; log rank test), significant p-values in bold (threshold p ≤ 0.05). Prognostic group IV has been removed due to n = 0.

Abbreviations: PCa = prostate cancer; ERα = Estrogen receptor α; TS = tumor associated stroma; TE = Tumor epithelium, CFFS = Clinical failure free survival; PSA = Prostate specific antigen; GS = Gleason score; TS = tumor stage; NE = No event.

**Table 4:** Cox regression analysis (backwards stepwise model) summarizing significant independent prognostic factors for BF, CF and PCD in PCa patients (n = 535), significant p values in bold (0.05 threshold).

Marker	BF (n = 170)		CF (n = 36)		PCD (n = 15)	
	HR (95% CI)	p	HR (95% CI)	p	HR (95% CI)	p
pT - stage		**<0.001**				
pT2	1		NE		NE	
pT3a	1.81 (1.22–2.63)	**0.003**				
pT3b	2.84 (1.74–4.65)	**<0.001**				
Gleason grade		0.055		**0.019**		0.085
3 + 3	1		1		1	
3 + 4	1.02 (0.68–1.51)	0.922	2.12 (0.74–1.20)	0.160	3.55 (0.39–32.03)	0.26
4 + 3	1.45 (0.90–2.30)	0.127	3.00 (0.94–9.56)	0.063	9.05 (1.02–80.52)	0.048
4 + 4	1.28 (0.61–2.70)	0.513	2.89 (0.55–15.14)	0.210	5.97 (0.36–100.39)	0.22
≥9	2.27 (1.25–4.12)	**0.007**	6.80 (2.17–21.32)	**0.001**	15.67 (1.70–144.62)	**0.015**
PNI	NE		2.12 (1.03–4.39)	**0.043**	3.4 (1.1–10.53)	**0.034**
Preop. PSA	1.37 (0.99–1.91)	0.057	NE		NE	
Apical PSM	0.69 (0.49–0.98)	**0.038**	NE		NE	
Non-apical PSM	1.72 (1.21–2.44)	**0.002**	3.16 (1.52–6.60)	**0.002**	NE	
ERβ TS	1.70 (1.19–2.42)	**0.004**	NE		NE	
Aromatase TS	0.55 (0.38–0.80)	**0.002**	NE		NE	
Aromatase TE	NE		0.43 (0.21–0.90)	**0.024**	0.33 (0.10–1.04)	0.059
ERα TS	NE		0.43 (0.22–0.87)	**0.018**	0.28 (0.10–0.78)	**0.015**

Abbreviations: ERα = estrogen receptor alpha; ERβ = estrogen receptor beta; TS = tumor associated stromal cells; TE = tumor epithelial cells; BF = biochemical failure; CF = clinical failure; PCD = prostate cancer death; PNI = perineural infiltration; PSA = prostate specific antigen; PSM = positive surgical margin; NE = nor entered.
