# Supplementary material for: Estrogen receptors α and β and aromatase as independent predictors for prostate cancer outcome
Source: Sci Rep. 2016 Sep 9;6:33114. doi: 10.1038/srep33114 (PMC5017140; doi:10.1038/srep33114)
Supplement: Supplementary Information [file srep33114-s1.pdf]

Supplementary information

Estrogen receptors α and β and aromatase as independent predictors for prostate cancer outcome

Thea Grindstad[1], Kaja Skjefstad[1], Sigve Andersen MD, PhD[2,3], Nora Ness[1], Yngve Nordby MD[2], Samer Al-Saad MD, PhD[1,4], Silje Fismen MD, PhD[4], Tom Donnem, MD, PhD[2,3], Mehrdad Rakaee khanehkenari[1], Lill-Tove Busund, MD, PhD[1,4], Roy M. Bremnes, MD, PhD[1,2], Elin Richardsen, MD, PhD[1,4]

[1]Dept. of Medical Biology, UiT The Arctic University of Norway, Tromso, Norway

[2]Dept. of Clinical Medicine, UiT The Arctic University of Norway, Tromso, Norway

[3]Dept. of Oncology, University Hospital of North Norway, Tromso, Norway

[4]Dept. of Clinical Pathology, University Hospital of North Norway, Tromso, Norway

Corresponding author and reprints:

Thea Grindstad

IMB - Dept. of Medical Biology

UiT The Arctic University of Norway

9019 Tromso, Norway

Telephone +47 95878050

Fax: +47 77672704

E-mail: tgr015@post.uit.no

Antibody Validation

Cell lines

Six human lung, prostate and breast cancer cell lines A549 (CCL-185), NCI-H460 (HTB-177), DU145 (HTB-81), PC3 (CRL-1435), MCF7(HTB-22) and MDA-MB-231(HTB-26), all from ATCC (Manassas, VA), were cultured in RPMI 1640 media (R8758, Sigma-Aldrich). All media were supplemented with 10% fetal bovine serum (S0415, Biochrom) and 1× penicillin-streptomycin antibiotic mixture (P0781, Sigma-Aldrich). Cells were incubated at 37°C in a humidified atmosphere with 5% CO_2. STR-profiling was performed to verify the cell lines authenticity by the department of Forensic Medicine at UiT The Arctic University of Norway.

Western Blot analysis

Cells were washed in ice-cold phosphate-buffered saline, and lysate was added directly in NuPAGE LDS Sample Buffer (NP0007, Life Technologies) with dithiothreitol. In addition, HEK 293 cell lysates were utilized beside from OriGene for ERα (LY400046), ERβ1 (LY425704) and aromatase (LY400031) and were incubated with 2xSDS Sample Buffer (OriGene) for 10 minutes at 100°C. Equal amounts of protein lysates were resolved onto a 4% to 12% Bis-Tris gel (NP0322; Life Technologies). The resolved proteins were transferred onto an Odyssey nitrocellulose membrane (926-31092, LI-COR), and the membrane was subsequently blocked for 1 hour at room temperature using the Odyssey blocking buffer (927-40000, LI-COR). For all three Primary antibodies 1/500 dilution applied and the membrane incubated for over night at 4˚C. The following IRDye 800CW secondary antibodies for ERα (926-32213, LI-COR),ERβ1 (926-32212, L1-COR) and aromatase (935-32214,L1-COR) with 1/10000 dilution incubated 1 hour at RT. Rabbit anti-actin, 1:1000 (A2066, Sigma-Aldrich)

was used as internal control and all lanes shows 42 KDa molecular weight protein load. Between antibody incubations, the membrane was washed three times for 5 minutes each time in tris-buffered saline containing 0.05 % Tween 20 (Sigma-Aldrich). Molecular weight markers used were the MagicMark XP Western Protein Standard (LC5603, Invitrogen) and SeeBlue Plus2 Pre-stained Standard (LC5925, Invitrogen).

Antibody validation results

Western blot analysis was used to verify the specificity of the primary antibodies (Figure S1, Table S1). The observed molecular weight of the detected protein (the most prominent bands) in lung, prostate and breast cell line lysates corresponded intimately with the predicted weight, as with the data provided by the manufacturers. Transiently overexpressed lysate of each primary antibody and empty vector was applied in order to further accredit specificity. Minor variations of molecular weight band location can be explained by specific conditions during sample preparation and post-translational modification. This includes glycosylation and phosphorylation, which can result in an increase in molecular weight.

Supplementary figure 1

Western blot analysis verifying the specificity of the primary antibodies (A, C, E) using rabbit actin as internal control (B, D, F): A-B) ERα, C-D) ERβ and E-F) aromatase. Six human cancer cell liens including lung (A549, NCI-H460), prostate (DU145, PC3) and breast cancer (MCF7 and MDA-MB-231), corresponding over-expressed domain protein lysates (HEK293T) and negative control of over-expressed lysates (HEK293) were applied. The most prominent bands represent the observed molecular weight of the detected protein, which corresponded intimately with the predicted weight provided by the manufacturer.

A

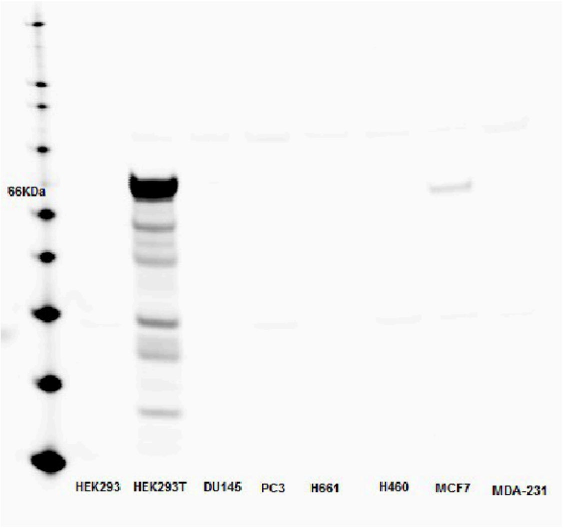
66KDa
HEK293
HEK293T
DU145
PC3
H661
H460
MCF7
MDA-231

B

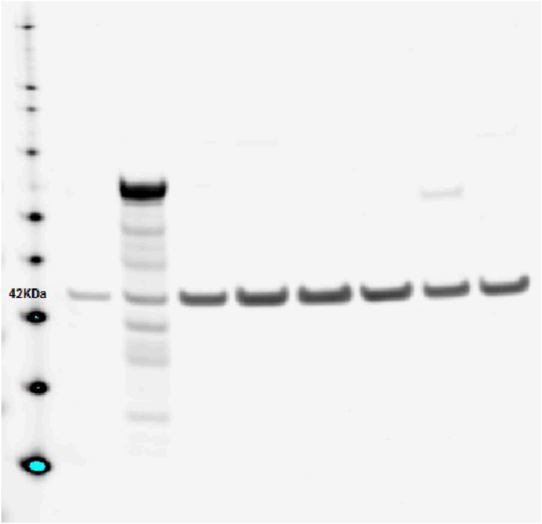
42KDa

C

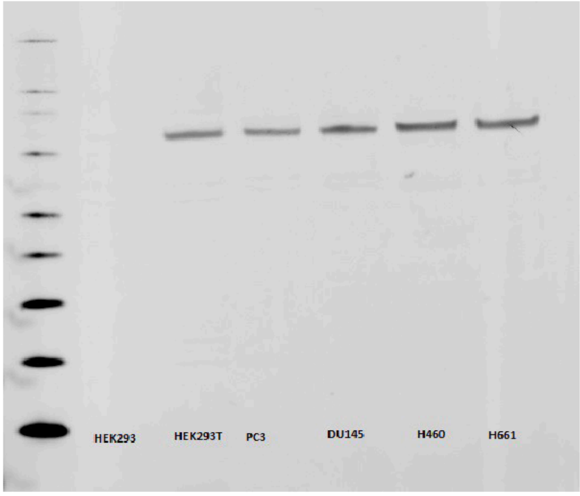
HEK293
HEK293T
PC3
DU145
H460
H661

D

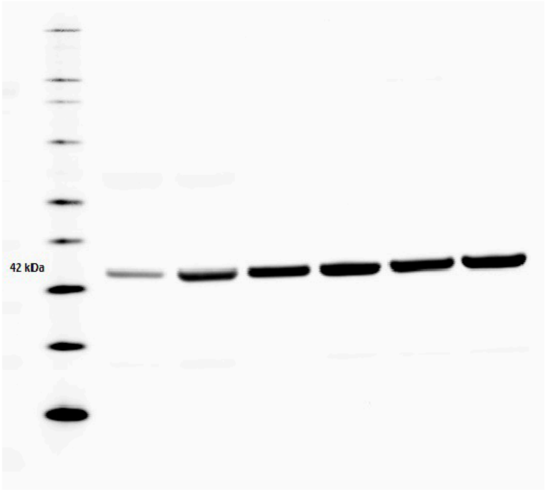
42 kDa

E

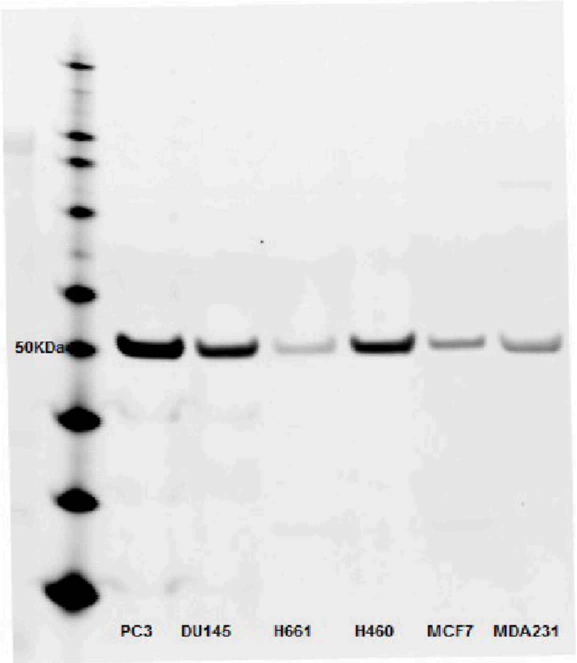
50KDa
PC3
DU145
H661
H460
MCF7
MDA231

F

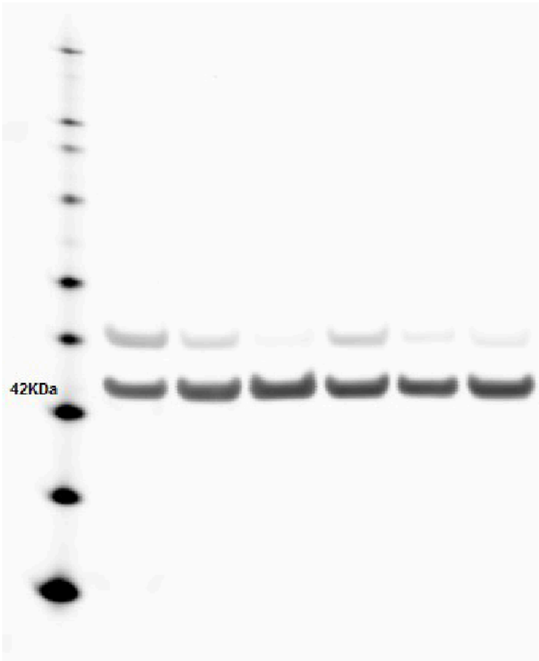
42KDa

Supplementary table 1

Table illustrating results from antibody validation using western blot analysis.

	HEK293 (Neg. control)	HEK293T	Predicted MW	Observed MW	H460	A549	PC3	DU145	MCF7	MDA-MB-231 (Triple Neg)	Predicted MW(KDa)	Observed MW(Kda)
**ERα**	-	+	66	66	-	-	-	-	+	-	66	66
**ERβ**	-	+	55.3	≃ 70	+	+	+	+	n.a	n.a	53-58	≃ 70
**Aromatase**	n.a.	n.a.	n.a.	n.a.	+	+	+	+	+	+	58	54
**Actin**	+	+	42	42	+	+	+	+	+	+	42	42

Abbreviations: ER = estrogen receptor; MW = molecular weight; n.a. = not applied

Supplementary table 2

Combined marker expressions as predictor for BFFS, CFFS and PCDFS in PCa patients (n = 535), (univariate analysis; log rank test), significant p-values in bold (threshold $p \leq 0.05$)

**Marker expression**		**Patients (n)**	**Patients (%)**	**BFFS**			**CFFS**			**PCDFS**		
				**5-year (%)**	**10-year (%)**	**p**	**5-year (%)**	**10-year (%)**	**p**	**5-year (%)**	**10-year (%)**	**p**
**ERα + Aromatase TS**	Low	213	39.8	70	61	0.426	94	87	**0.029**	99	96	0.154
	High	303	56.8	76	63		98	96		99	98	
	Missing	18	3.4									
**ERβ + Aromatase TS**	Low/low	117	21.9	69	56	**>0.001**			NS			NS
	High/low	14	2,3	47	31							
	Low/high	251	50.0	81	70							
	High/high	135	25.2	69	56							
	Missing	18	3.4									
**ERα TS + Aromatase TE**	Low	117	21.9			**NS**	93	80	**0.038**	98	93	**0.003**
	High	399	74.6				97	92		99	98	
	Missing	19	3.5									
**ERβ TS + Aromatase TE**	Low	218	40.7			NS			NS			NS
	High	299	55.9									
	Missing	18	3.4									

Abbreviations: PCa = prostate cancer; ERα = estrogen receptor alpha; ERβ = estrogen receptor beta; TE = tumor epithelial cells; TS = tumor stromal cells; BFFS = Biochemical failure free survival; CFFS = clinical failure free survival; PCDFS = prostate cancer death free survival; NS: Not significant
